# Temporal changes in laboratory markers of survivors and non-survivors of adult inpatients with COVID-19

**DOI:** 10.1186/s12879-020-05678-0

**Published:** 2020-12-11

**Authors:** Song-Mao Ouyang, Hong-Quan Zhu, Ying-Na Xie, Zhi-Sheng Zou, Hui-Min Zuo, Yun-Wei Rao, Xiao-Yan Liu, Bin Zhong, Xin Chen

**Affiliations:** 1 Department of Intensive Medicine, First Affiliated Hospital of Gannan Medical University, Gannan Medical University, Ganzhou, 341000 China; 2 Department of Pathogenic Biology, School of Basic Medical Sciences, Gannan Medical University, Ganzhou, 341000 China; 3 Department of Respiratory, First Affiliated Hospital of Gannan Medical University, Gannan Medical University, Ganzhou, 341000 China; 4 Department of Pharmacy, First Affiliated Hospital of Gannan Medical University, Gannan Medical University, Ganzhou, 341000 China

**Keywords:** COVID-19, SARS-CoV − 2, Clinical characteristics, Temporal changes, Laboratory tests

## Abstract

**Background:**

Coronavirus disease 2019 (COVID-19) is caused by severe acute respiratory syndrome coronavirus 2, and outbreaks have occurred worldwide. Laboratory test results are an important basis for clinicians to determine patient condition and formulate treatment plans.

**Methods:**

Fifty-two thousand six hundred forty-four laboratory test results with continuous values of adult inpatients who were diagnosed with COVID-19 and hospitalized in the Fifth Hospital in Wuhan between 16 January 2020 and 18 March 2020 were compiled. The first and last test results were compared between survivors and non-survivors with variance test or Welch test. Laboratory test variables with significant differences were then included in the temporal change analysis.

**Results:**

Among 94 laboratory test variables in 82 survivors and 25 non-survivors with COVID-19, white blood cell count, neutrophil count/percentage, mean platelet volume, platelet distribution width, platelet-large cell percentage, hypersensitive C-reactive protein, procalcitonin, D-dimer, fibrin (ogen) degradation product, middle fluorescent reticulocyte percentage, immature reticulocyte fraction, lactate dehydrogenase were significantly increased (*P* < 0.05), and lymphocyte count/percentage, monocyte percentage, eosinophil percentage, prothrombin activity, low fluorescent reticulocyte percentage, plasma carbon dioxide, total calcium, prealbumin, total protein, albumin, albumin-globulin ratio, cholinesterase, total cholesterol, nonhigh-density/low-density/small-dense-low-density lipoprotein cholesterol were significantly decreased in non-survivors compared with survivors (*P* < 0.05), in both first and last tests. Prothrombin time, prothrombin international normalized ratio, nucleated red blood cell count/percentage, high fluorescent reticulocyte percentage, plasma uric acid, plasma urea nitrogen, cystatin C, sodium, phosphorus, magnesium, myoglobin, creatine kinase (isoenzymes), aspartate aminotransferase, alkaline phosphatase, glucose, triglyceride were significantly increased (*P* < 0.05), and eosinophil count, basophil percentage, platelet count, thrombocytocrit, antithrombin III, red blood cell count, haemoglobin, haematocrit, total carbon dioxide, acidity-basicity, actual bicarbonate radical, base excess in the extracellular fluid compartment, estimated glomerular filtration rate, high-density lipoprotein cholesterol, apolipoprotein A1/ B were significantly decreased in non-survivors compared with survivors (*P* < 0.05), only in the last tests. Temporal changes in 26 variables, such as lymphocyte count/percentage, neutrophil count/percentage, and platelet count, were obviously different between survivors and non-survivors.

**Conclusions:**

By the comprehensive usage of the laboratory markers with different temporal changes, patients with a high risk of COVID-19-associated death or progression from mild to severe disease might be identified, allowing for timely targeted treatment.

**Supplementary Information:**

The online version contains supplementary material available at 10.1186/s12879-020-05678-0.

## Background

In December 2019, an outbreak of coronavirus disease 2019 (COVID-19), caused by severe acute respiratory syndrome coronavirus 2 (SARS-CoV-2), started in Wuhan city, the capital of the Hubei Province in China [1, 2]. According to the report of World Health Organization, as of 3 November 2020, there were 46,840,783 patients with confirmed COVID-19 worldwide, and 1,204,028 of them died [3]. The number of confirmed cases has increased by more than 400,000 per day.

The most common clinical manifestations of COVID-19 are fever, cough, shortness of breath, muscle aches, confusion, and headache [4]. Studies revealed that prothrombin time (PT), glucose level, hypersensitive C-reactive protein (hs-CRP), procalcitonin (PCT), interleukin-6 (IL-6), and fibrinogen were above the normal reference range, while haemoglobin was below the normal reference range [5–9]. Analysis of systematic laboratory test results might deepen our understanding of the disorders caused by COVID-19.

The causes of death of COVID-19 include respiratory failure, heart failure, renal failure, liver failure and other reasons [10]. Articles reported that elevated odds of inpatient death were associated with older age, higher sequential organ failure assessment and D-dimer greater than 1 μg/mL on admission [11]. Studies also revealed that cytokine storm might be one of the main causes of COVID-19 associated death, including the decrease in total lymphocytes and lymphocyte subsets, and the elevation of C-reactive protein, erythrocyte sedimentation rate, serum amyloid, PCT, ferritin, cytokines [12, 13]. Although these findings were useful in identifying patients at high risk of death, more intuitive markers are urgently needed. In addition, severe patients had a much higher risk of death than non-severe patients [4, 5]. Intuitive markers that might indicate the progression from non-severe to severe disease are also urgently needed.

A better understanding of the pathogenesis of COVID-19 and a series of intuitive markers to identify patients with high odds of death would provide useful guidelines to reduce the mortality of COVID-19. To achieve this goal, we compiled the laboratory test results and explored the temporal changes in laboratory makers with continuous values of survivors and non-survivors with COVID-19 in Wuhan, China.

## Methods

### Participants and data collection

One hundred seven inpatients (≥ 18 years old) who were diagnosed as SARS-CoV-2 nucleic acid-positive by real-time polymerase chain reaction and hospitalized in the Fifth Hospital in Wuhan (Wuhan, China) between 16 January 2020 and 18 March 2020 were included in this retrospective study. The demographic, clinical, and laboratory data of these inpatients were extracted from electronic records by three physicians (Xiao-Yan Liu, Yun-Wei Rao, and Hui-Min Zuo) and checked by three other physicians (Zhi-Sheng Zou, Hong-Quan Zhu and Song-Mao Ouyang). For the purpose of exploring temporal changes in laboratory markers, only laboratory test results with continuous values were included in the following analyses.

### Definitions

All the participants with COVID-19 were divided into two groups: survivors and non-survivors. Participants who met the following criteria were defined as survivors: had two consecutive negative nucleic acid tests for SARS-CoV-2 and were discharged; had two consecutive negative nucleic acid tests for SARS-CoV-2 and were transferred to a designated hospital for further treatment; or had a positive nucleic acid test for SARS-CoV-2 and were transferred to designated hospitals for further treatment by the Prevention and Control Emergency Command of COVID-19 in Hubei Province due to the presence of improved symptoms after treatment or the closure of this emergency hospital. Participants who infected with SARS-CoV-2 and died by 18 March 2020 were defined as non-survivors. According to this criterion, 82 participants were defined as survivors and 25 participants were defined as non-survivors.

### Statistical analysis

To explore the temporal changes in laboratory markers, only variables with continuous values were included in this analysis. If the test result was greater or less than the detection sensitivity, it was replaced with the upper or lower limit of the detection sensitivity. Each patient was tested only once a day. If a patient had tested more than once on a given day, the average value of all test results was used as the test result for that day. As the duration of hospital stay and the number of detection times varied greatly, we used the first and last test results to approximate the admission and discharge/mortality results. The average values of the first test and last test were compared between survivors and non-survivors using the variance test or Welch test, while appropriate.

**Fig. 1:**
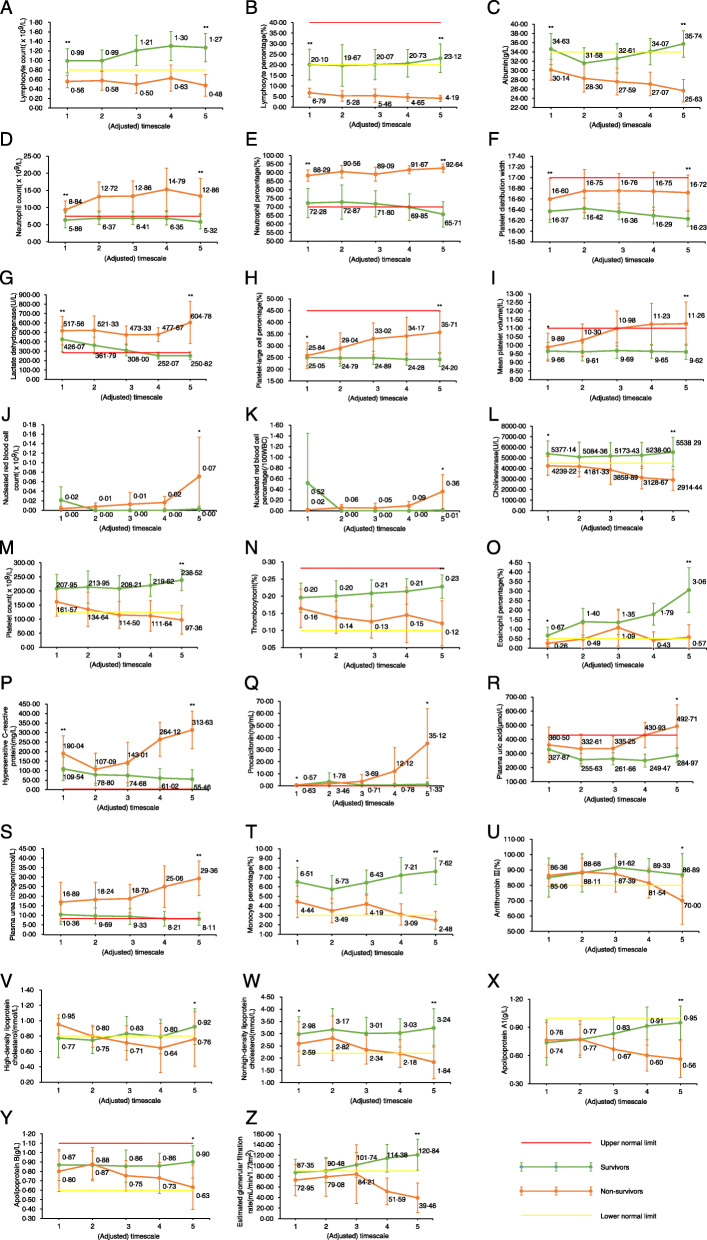
Temporal changes of laboratory makers of adult inpatients with COVID-19 in Wuhan, China. Laboratory test results of each time point is showed in average with 95% confidence intervals. If the lower 95% confidence interval is less than zero, it was replaced with zero. *, ** indicate that the *P* value of variance/welch test in survivors and non-survivors are between 0.001 and 0.05, less than 0.001 in the first test (above the first timescale point), and the last test (above the last timescale point), respectively

Temporal changes in the laboratory makers with significant differences between survivors and non-survivors in first and/or last test were explored. Comprehensively considering the impact of the number of tests and the sample size, only variables that were assessed five times or more were included in this analysis. Since the detection times of different variables varied greatly, quartiles were used to divide all the detection results and adjust the timescales of temporal changes for this analysis (Table S1, Fig. 1 and Fig. S1).

All statistical analyses were performed using the statistical software Statistical Package for Social Sciences (SPSS, version 22.0, IBM Corporation, Armonk, USA), and a two-sided *P*-value less than 0.05 was considered statistically significant.

## Results

A total of 107 inpatients were included in this retrospective analysis, with an average age of 57.5 years. Among them, 82 were defined as survivors, and 25 were defined as non-survivors; 64 were male, and 43 were female. The average age of non-survivors was significantly higher than that of survivors (63.5 years versus 55.7 years, *P* = 0.018).

The laboratory test variables of white blood cells and platelets of survivors were in the normal reference range as well (Table 1). While the white blood cell count (WBC) and neutrophil count/percentage of non-survivors were above the normal reference range, the lymphocyte count/percentage and eosinophil percentage of non-survivors were below the normal reference range. In addition, lymphocyte count/percentage, eosinophil count/percentage, monocyte percentage, basophil percentage, platelet count, and thrombocytocrit were significantly lower in non-survivors than in survivors, while WBC, neutrophil count/percentage, mean platelet volume (MPV), platelet distribution width (PDW), and platelet-large cell percentage were significantly higher in non-survivors than in survivors, implying that bacterial coinfection and hypercoagulability were important causes of COVID-19 patient death.

**Table 1:** The laboratory test results of adult inpatients with COVID-19 in Wuhan, China

Laboratory markers	Normal reference range	First tests			Last tests		
		Survivors	Non-survivors	*P* value	Survivors	Non-survivors	*P* value
WBC, ×10^9^/L	3.5–9.5	6.22	10.86 ↑	< 0.001 **	6.34	14.72 ↑	< 0.001 **
Lymphocyte count, ×10^9^/L	0.8–4	1.04	0.62 ↓	< 0.001 **	1.33	0.55 ↓	< 0.001 **
Lymphocyte percentage, %	20–40	20.82	6.70 ↓	< 0.001 **	24.31	4.56 ↓	< 0.001 **
Neutrophil count, ×10^9^/L	2–7	4.74	9.77 ↑	< 0.001 **	4.39	13.65 ↑	< 0.001 **
Neutrophil percentage, %	50–70	71.39 ↑	88.14 ↑	< 0.001 **	65.22	91.31 ↑	< 0.001 **
Monocyte count, ×10^9^/L	0.12–1.2	0.37	0.41	0.457	0.45	0.40	0.378
Monocyte percentage, %	3–12	6.53	4.47	0.002 *	7.62	3.27	< 0.001 **
Eosinophil count, ×10^9^/L	0.02–0.5	0.05	0.03	0.292	0.13	0.05	0.001 *
Eosinophil percentage, %	0.5–5	0.88	0.42 ↓	0.015 *	2.34	0.63	< 0.001 **
Basophil count, ×10^9^/L	0–0.1	0.02	0.03	0.35	0.03	0.03	0.682
Basophil percentage, %	0–1	0.37	0.27	0.152	0.50	0.28	0.008 *
Platelet count, × 10^9^/L	125–350	214.66	178.77	0.1	232.63	125.48	< 0.001 **
MPV, fL	7–11	9.23	10.01	0.003 *	9.20	11.11 ↑	< 0.001 **
PDW	15–17	16.18	16.63	< 0.001 **	16.14	16.74	< 0.001 **
Thrombocytocrit, %	0.1–0.282	0.19	0.18	0.41	0.21	0.14	< 0.001 **
Platelet-large cell count, ×10^9^/L	30–90	43.45	45.79	0.606	47.80	39.58	0.066
Platelet-large cell percentage, %	11–45	21.60	26.75	0.003 *	21.22	34.67	< 0.001 **
hs-CRP, mg/L	< 3	59.58 ↑	191.76 ↑	< 0.001 **	22.36 ↑	248.49 ↑	< 0.001 **
PCT, ng/mL	0–0.05	0.30 ↑	0.89 ↑	0.019 *	0.28 ↑	23.36 ↑	0.009 *
IL-6, pg/ml	0–7	40.65 ↑	76.10 ↑	0.137	111.77 ↑	1012.76 ↑	0.13
PT, s	11–15	15.58 ↑	23.15 ↑	0.089	14.76	19.57 ↑	0.002 *
PT-INR	0.8–1.2	1.22 ↑	2.19 ↑	0.132	1.14	1.65 ↑	0.003 *
PTA, %	80–150	78.48 ↓	52.83 ↓	< 0.001 **	90.39	57.67 ↓	< 0.001 **
TT, s	13–20	20.17 ↑	19.64	0.864	17.59	18.94	0.139
aPTT, s	28–44	42.52	46.61 ↑	0.285	41.62	47.07 ↑	0.245
Fibrinogen, g/L	2–4	4.38 ↑	5.07 ↑	0.098	4.13 ↑	4.54 ↑	0.248
D-dimer, μg/mL	0–0.5	2.83 ↑	9.92 ↑	0.002 *	1.58 ↑	12.24 ↑	< 0.001 **
FDP, μg/mL	0–5	15.55 ↑	86.68 ↑	0.014 *	7.32 ↑	75.67 ↑	< 0.001 **
Antithrombin III, %	80–120	91.71	86.19	0.179	95.97	75.21 ↓	0.001 *
RBC, × 10^12^	3.5–5.5	4.11	3.86	0.143	3.80	3.21 ↓	0.003 *
Haemoglobin #, g/L	124.2–165.3	122.34 ↓	115.27 ↓	0.164	111.85 ↓	94.73 ↓	0.005 *
Haematocrit, %	40–54	37.97 ↓	36.06 ↓	0.217	35.37 ↓	30.31 ↓	0.006 *
MCV, fL	82–100	92.61	92.89	0.827	93.41	94.43	0.412
MCH, pg	27–34	29.66	29.89	0.621	29.49	29.36	0.775
MCHC, g/L	316–354	320.24	321.52	0.574	315.61 ↓	310.94 ↓	0.109
RDW-CV, %	11.5–14.5	13.04	12.93	0.618	13.65	13.44	0.471
RDW-SD, fL	35–56	42.21	41.66	0.474	44.40	44.00	0.689
nRBC, ×10^9^/L		0.01 ↑	0.01 ↑	0.981	0	0.06 ↑	0.033 *
nRBC%, /100WBC		0.15 ↑	0.04 ↑	0.632	0	0.29 ↑	0.011 *
Ret, ×10^12^	0.02–0.2	0.04	0.05	0.488	0.07	0.06	0.208
Ret%, %	0.3–3	1.09	1.31	0.133	1.98	1.89	0.793
lf-Ret%, %	80–100	93.13	90.54	0.035 *	91.54	87.72	0.004 *
mf-Ret%, %	0–20	6.38	8.93	0.011 *	7.96	10.71	0.009 *
hf-Ret%, %	0–5	0.49	0.53	0.893	0.51	1.57	0.025 *
IRF, %	0–25	6.87	9.46	0.035 *	8.46	12.28	0.004 *
SpO_2_, %	95–98	90.35 ↓	90.72 ↓	0.926	94.89 ↓	87.54 ↓	0.119
TCO_2_, mmol/L	22–29	21.85 ↓	19.60 ↓	0.333	27.88	23.48	0.002 *
PaO_2_, mmHg	80–105	96.64	76.10 ↓	0.205	101.79	87.55	0.351
PaCO_2_, mmHg	35–45	33.51 ↓	28.45 ↓	0.095	45.16 ↑	48.43 ↑	0.539
Acidity-basicity	7.35–7.45	7.40	7.44	0.268	7.39	7.31 ↓	0.035 *
ABR, mmol/L	22–27	20.82 ↓	18.72 ↓	0.351	26.49	22.01	0.001 *
BEEcf, mmol/L	-2-3	−3.43 ↓	−4.95 ↓	0.529	1.96	−3.51 ↓	0.001 *
PCO_2_, mmol/L	22–29	20.57 ↓	17.58 ↓	0.009 *	22.36	19.40 ↓	0.002 *
PUA, μmol/L	210–430	330.99	372.57	0.5	290.85	454.43 ↑	0.007 *
Plasma creatinine, μmol/L	53–123	322.79 ↑	459.80 ↑	0.303	236.91 ↑	389.64 ↑	0.081
PUN, mmol/L	2.9–8.2	11.01 ↑	17.86 ↑	0.093	9.12 ↑	25.45 ↑	< 0.001 **
eGFR, mL/min/1.73m^2^	> 90	87.85 ↓	69.88 ↓	0.168	101.30 ↑	46.13 ↓	< 0.001 **
Cystatin C, mg/L	0–1.5	2.48 ↑	3.28 ↑	0.24	2.38 ↑	3.67 ↑	0.03 *
Sodium, mmol/L	135–145	139.43	140.32	0.39	139.85	144.40	0.034 *
Potassium, mmol/L	3.5–5.5	4.06	4.11	0.828	4.25	4.67	0.096
Total calcium, mmol/L	2.08–2.6	2.10	1.97 ↓	0.027 *	2.19	1.94 ↓	< 0.001 **
Phosphorus, mmol/L	1–1.6	1.23	1.50	0.234	1.27	1.69 ↑	0.029 *
Magnesium, mmol/L	0.6–1.1	0.97	1.01	0.562	0.90	1.02	< 0.001 **
Chlorine, mmol/L	95–110	103.52	104.18	0.695	104.00	105.77	0.437
hs-cTnI, ng/mL	0–0.023	0.03 ↑	0.01	0.313	0.02	0.37 ↑	0.166
Myoglobin, ng/mL	23–112	195.78 ↑	342.42 ↑	0.126	233.33 ↑	547.84 ↑	0.005 *
NT-proBNP, ng/L	0–900	5179.35 ↑	6173.35 ↑	0.784	6204.18 ↑	12,539.35 ↑	0.156
LDH, U/L	80–285	276.57	465.15 ↑	< 0.001 **	203.73	602.65 ↑	< 0.001 **
HCA, μmol/L	0–15	12.96	10.02	0.242	14.23	12.81	0.606
CK, U/L	50–310	130.39	373.00 ↑	0.192	70.34	402.55 ↑	0.006 *
CKI, ng/mL	0–7.2	8.15 ↑	5.60	0.466	3.84	25.03 ↑	0.041 *
Total bilirubin, μmol/L	0–25	13.44	17.45	0.351	14.89	21.59	0.292
Direct bilirubin, μmol/L	0–6.84	6.98 ↑	8.09 ↑	0.758	5.82	14.27 ↑	0.09
Total bile acid, μmol/L	0–10	11.10 ↑	5.44	0.51	5.16	7.78	0.276
Prealbumin, mg/L	160–450	179.61	87.87 ↓	< 0.001 **	224.84	98.51 ↓	< 0.001 **
Total protein, g/L	60–85	70.26	65.30	0.01 *	72.05	62.88	0.002 *
Albumin, g/L	34–55	36.60	30.27 ↓	< 0.001 **	36.64	26.99 ↓	< 0.001 **
Globulin, g/L	20–40	33.65	35.04	0.304	35.40	35.89	0.795
AGR	1.2–2.4	1.11 ↓	0.89 ↓	0.001 *	1.08 ↓	0.80 ↓	< 0.001 **
ALT, U/L	9–50	32.19	34.57	0.741	27.47	46.62	0.202
AST, U/L	15–40	34.49	47.91 ↑	0.065	26.75	82.59 ↑	0.015 *
ALP, U/L	45–125	64.30	77.17	0.243	68.48	98.29	0.004 *
GGT, U/L	10–60	52.87	64.15 ↑	0.511	45.22	51.70	0.476
Cholinesterase, U/L	4500–12,000	5844.80	4521.15	0.003 *	5849.76	3611.55 ↓	< 0.001 **
Glucose, mmol/L	3.9–6.1	6.93 ↑	8.11 ↑	0.22	6.04	8.39 ↑	< 0.001 **
Triglyceride, mmol/L	< 2.3	1.67	1.45	0.527	1.65	2.15	0.044 *
Total cholesterol, mmol/L	0–5.1	3.92	3.26	0.015 *	4.34	2.92	< 0.001 **
HDL-C, mmol/L	0.8–2	0.95	0.86	0.323	1.07	0.79 ↓	0.006 *
non-HDL-C, mmol/L	2.2–5.8	2.98	2.40	0.029 *	3.27	2.13 ↓	< 0.001 **
LDL-C, mmol/L	0–3.36	2.46	1.91	0.021 *	2.80	1.50	< 0.001 **
sdLDL-C, mmol/L	0.23–1.37	0.73	0.48	0.028 *	0.92	0.47	0.001 *
Apolipoprotein A1, g/L	1–1.6	0.90 ↓	0.76 ↓	0.057	1.07	0.61 ↓	< 0.001 **
Apolipoprotein B, g/L	0.6–1.1	0.84	0.73	0.087	0.87	0.68	0.006 *
Lipoprotein(a), mg/L	0–300	185.60	189.68	0.938	208.12	133.05	0.129

^↑^ above normal reference range. ^↓^ below normal reference range. ^*^ *P* value less than 0.05 and more than 0.001. ^**^ *P* value less than 0.001. ^#^ The normal reference range of haemoglobin was calculated based on the number of female and male, cause the normal reference range of haemoglobin of female was 115–150 g/L, the normal reference range of haemoglobin of male was 130–175 g/L. *ABR* Actual bicarbonate radical; *AGR* Albumin-globulin ratio; *ALP* Alkaline phosphatase; *ALT* Alanine aminotransferase; *aPTT* Activated partial thromboplastin time; *AST* Aspartate aminotransferase; *BEEcf* Base excess in the extracellular fluid compartment; *CK* Creatine kinase; *CKI* Creatine kinase isoenzymes; *eGFR* Estimated glomerular filtration rate; *FDP* Fibrin (ogen) degradation product; *GGT* γ-Glutamyl transferase; *HCA* Homocysteic acid; *HDL-C* High-density lipoprotein cholesterol; *hf-Ret%* High fluorescent reticulocyte percentage; *hs-CRP* Hypersensitive C-reactive protein; *hs-cTnI* High-sensitivity cardiac troponin I; *IL-6* Interleukin-6; *IRF* Immature reticulocyte fraction; *LDH* Lactate dehydrogenase; *LDL-C* Low-density lipoprotein cholesterol; *lf-Ret%* Low fluorescent reticulocyte percentage; *MCH* Mean corpuscular volume; *MCHC* Mean corpuscular haemoglobin concentration; *MCV* Mean corpuscular volume; *mf-Ret%* Middle fluorescent reticulocyte percentage; *MPV* Mean platelet volume; *non-HDL-C* Nonhigh-density lipoprotein cholesterol; *nRBC* Nucleated red blood cell count; *nRBC%* Nucleated red blood cell percentage; *NT-proBNP* N-terminal pro B-type natriuretic peptide; *PaCO*_*2*_ Partial pressure of carbon dioxide; *PaO*_*2*_ Partial pressure of oxygen; *PCO*_*2*_ Plasma carbon dioxide; *PCT* Procalcitonin; *PDW* Platelet distribution width; *PT* Prothrombin time; *PTA* Prothrombin activity; *PT-INR* Prothrombin international normalized ratio; *PUA* Plasma uric acid; *PUN* Plasma urea nitrogen; *RBC* Red blood cell count; *RDW-CV* Red blood cell distribution width - coefficient of variation; *RDW-SD* Red blood cell distribution width - standard deviation; *Ret* Reticulocyte count; *Ret%* Reticulocyte percentage; *sdLDL-C* Small-dense low-density lipoprotein cholesterol; *SpO*_*2*_ Oxygen saturation; *TCO*_*2*_ Total carbon dioxide; *TT* Thrombin time; *WBC* White blood cell count

After SARS-CoV-2 infection, hs-CRP, PCT, IL-6, PT, fibrinogen, D-dimer, fibrin (ogen) degradation product (FDP), nucleated red blood cell count (nRBC), nucleated red blood cell percentage (nRBC%) were higher than the upper normal limit, while prothrombin activity (PTA), haemoglobin, haematocrit, oxygen saturation (SpO_2_), total carbon dioxide (TCO_2_), partial pressure of oxygen (PaO_2_), blood pH, actual bicarbonate radical (ABR), and base excess in the extracellular fluid compartment (BEEcf) were lower than the lower normal limit, both in survivors and non-survivors (Table 1). Compared with those in survivors, these increase/decrease were more serious in non-survivors, indicating that excessive systemic inflammation, hypercoagulability, and anaemia were the main symptoms of COVID-19 and risk factors for COVID-19-associated death.

In COVID-19 patients, plasma creatinine, plasma urea nitrogen (PUN), cystatin C, high-sensitivity cardiac troponin I, creatine kinase (CK), creatine kinase isoenzymes (CKI), myoglobin, lactate dehydrogenase (LDH), and N-terminal pro B-type natriuretic peptide (NT-proBNP) were higher than the upper normal limit, while the estimated glomerular filtration rate (eGFR) was lower than the lower normal limit (Table 1). Compared with those in survivors, these increases and decreases were more serious in non-survivors, indicating that the glomerular filtration rate and heart function were affected after SARS-CoV-2 infection and risk factors for COVID-19-associated death, especially glomerular filtration rate.

In survivors, total bilirubin, prealbumin, total protein, albumin, globulin, alanine aminotransferase, aspartate aminotransferase (AST), alkaline phosphatase (ASP), γ-glutamyl transferase, cholinesterase, triglyceride, cholesterol, apolipoprotein B, and lipoprotein(a) were normal, indicating that the liver might not be injured after SARS-CoV-2 infection (Table 1). In non-survivors, the prealbumin, albumin, albumin-globin ratio, cholinesterase, high-density/non-high-density lipoprotein cholesterol, and apolipoprotein A1 were below the normal reference range and significantly lower than that in survivors (*P* < 0.05), implying that a decline in liver synthesis function might be an important factor for COVID-19-associated death.

From the results of the temporal change analysis, lymphocyte count/percentage, albumin, neutrophil count/percentage, PDW, and LDH were significantly different throughout the course of the disease (Fig. 1), implying that these variables could be used as laboratory markers to distinguish COVID-19 patients with a high risk or low risk of infection-associated death at any timepoint during their treatment course (Fig. 1). Considering the lower and upper 95% confidence intervals of variables in survivors (Table S2), a lymphocyte count/percentage lower than 0.92 × 10^9^/L (17.77%), albumin lower than 35.14 g/L, neutrophil count/percentage higher than 5.51 × 10^9^ /L (74.83%), PDW higher than 16.28, or LDH higher than 314.55 U/L indicated a very high risk of death in COVID-19 patients.

The temporal change trends of platelet-large cell percentage, MPV, nRBC, nRBC%, cholinesterase, platelet count, thrombocytocrit, and eosinophil percentage were obviously different between survivors and non-survivors (Fig. 1), indicating that these variables could be used as laboratory markers to identify patients with a high risk of death during the course of the disease. The continuous rise in nRBC, nRBC%, MPV, and platelet-large cell percentage, the continuous drop in cholinesterase, platelet count, and thrombocytocrit, and the stagnation of eosinophil percentage at the lower normal limit (0.5%) meant an increasing risk of death in COVID-19 patients.

Compared with that in survivors, the temporal change in hs-CRP, PCT, plasma uric acid (PUA), and PUN in non-survivors had an obvious increase in the middle of the disease course, while monocyte percentage, antithrombin III, high-density lipoprotein cholesterol (HDL-C), nonhigh-density lipoprotein cholesterol (non-HDL-C), apolipoprotein A1, apolipoprotein B, and eGFR had an obvious decrease (Fig. 1), indicating that these variables could be used as laboratory markers to identify worsening COVID-19 during treatment. Increases of 39.0% in hs-CRP, 228.5% in PCT, 28.5% in PUA, and 34.0% in PUN, and decreases of 26.3% in monocyte percentage, 6.7% in antithrombin III, 15.8% in HDL-C, 17.0% in non-HDL-C, 13.0% in apolipoprotein A1, 14.8% in apolipoprotein B, and 38.7% in eGFR, indicated an increasing risk of death in COVID-19 patients.

The differences in the temporal changes in another 37 laboratory test variables between survivors and non-survivors were not as obvious (Fig. S1), but these variables were also useful for selecting patients with a high risk of COVID-19-associated death.

## Discussion

We performed a retrospective analysis of COVID-19 patients in Wuhan, China and revealed that in COVID-19 patients, PT, glucose level, hs-CRP, PCT, IL-6, and fibrinogen were above the normal reference range, while haemoglobin was below the normal reference range. These results were consistent with previous studies [5–9]. Previous studies also reported that COVID-19 patients had a higher level of LDH and a lower level of lymphocyte count/percentage and albumin than healthy individuals [5–9, 14]. Our study revealed that these variables were normal in survivors but were above/below the normal reference ranges in non-survivors. The level of classification might be the main reason for the above differences. In addition, we found that plasma creatinine, PUN, cystatin C, myoglobin, NT-proBNP, direct bilirubin, prothrombin international normalized ratio (PT-INR), D-dimer, FDP, and nucleated red blood count/percentage were higher and eGFR, albumin-globulin ratio (AGR), haematocrit, SpO_2_, TCO_2_, partial pressure of carbon dioxide, ABR, base excess in the extracellular fluid, plasma carbon dioxide (PCO_2_), apolipoprotein, and PTA were lower in COVID-19 patients. These results might deepen our understanding of the disorders caused by SARS-CoV-2.

The differences of temporal changes of laboratory test results are important markers for picking up severe patients after infecting SARS-CoV-2. Previous studies revealed that comparing with survivors, non-survivors had obvious increasing trends in D-dimer, IL-6, serum ferritin, high-sensitivity cardiac troponin, LDH, WBC, neutrophil count, blood urea nitrogen, and creatinine, and obvious decreasing trends in lymphocyte count [5, 11]. From systematic review and meta-analysis of laboratory test findings of COVID-19, Ghahramani et al. and Akbari et al. reported that cytokine storm might be one of the main causes of COVID-19 death [12, 13]. In the present study, the temporal changes of LDH, neutrophil count, blood urea nitrogen, and lymphocyte count were in accordance with the previous studies (Fig. 1). Additionally, compared with survivors, a significant increase in neutrophil percentage, platelet distribution with, platelet-large cell percentage, MPV, nucleated red blood cell, nucleated red blood cell percentage, high-sensitivity cardiac troponin I, PCT, and PUA, and a significant decrease in lymphocyte percentage, albumin, cholinesterase, platelet count, thrombocytocrit, eosinophil percentage, monocyte percentage, antithrombin III, high-density lipoprotein, cholesterol, non-HDL-C, apolipoprotein A1, apolipoprotein B, and estimated glomerular filtration were also observed (Fig. 1), implying that inflammation, coagulopathy, anaemia, and renal failure were also the main causes of COVID-19 death.

Although the clinical symptoms of COVID-19 include inflammation, coagulation dysfunction, anaemia, and renal failure, we speculated that tissue hypoxia might be one of the most important causes of death in COVID-19 patients. After being infected with SARS-CoV-2, patients have difficulty breathing and symptoms of pneumonia, which affect the patient’s oxygen partial pressure [9]. After the patient develops hypercoagulability, the blood flow through the tissue might decrease, which in turn increased tissue hypoxia [15]. Decreased haemoglobin reduces the efficiency of oxygen transport, resulting in tissue hypoxia as well. Tissue hypoxia could lead to disorders of multiple organs. Taking renal failure as an example, fulminating anoxia could lead to sympathetic nerve excitation and decreased glomerular plasma flow, which in turn lead to a reduction in glomerular filtration rate. During our treatment of COVID-19 patients, oliguria or anuria were found to be common in critically ill patients, indicating a reduction in glomerular plasma flow. This observation and laboratory test result of the eGFR (Table 1) might support our inference that tissue hypoxia might be the most important cause of death in COVID-19 patients.

To address the problem of hypoxia, clinicians have made many efforts. Oxygen therapy was the most common measure to improve the oxygen partial pressure of COVID-19 patients, including the use of invasive mechanical ventilation and extracorporeal membrane oxygenation that may lead to bacterial coinfection. Heparin sodium was used to treat hypercoagulability, and haemodialysis was used to treat renal failure in COVID-19 patients. However, tissue hypoxia still seemed to exist because cyanosis was common in severe COVID-19 patients who were dying. From the results of our study, PaO_2_ was normal at the last tests in both survivors and non-survivors (Table 1), probably due to the use of oxygen therapy. The haemoglobin and haematocrit were below the normal reference range, especially at the last tests in non-survivors. These results reflected that although the amount of dissolved oxygen in the plasma had improved, the oxygen transport capacity did not improve. In addition, the nRBC, nRBC% increased, especially in non-survivors, providing further proof of the absence of transport capacity. Therefore, although we did our best to improve the amount of dissolved oxygen in the plasma, the oxygen could not be fully used by tissues. Therefore, as the present trigger of red blood cell transfusion was with haemoglobin levels of 7–9 g/dL [16], we recommend adjusting this threshold in COVID-19 patients to improve their oxygen transport capacity. However, specific standards require more clinical trials to determine.

In the present study, reduced liver synthesis function and combined bacterial infection were found to be risk factors for COVID-19-associated death as well. For patients with high-risk factors, reasonable protein supplementation, careful choice of oxygen therapy, and appropriate antibacterial treatment should be considered during their treatment.

In addition to targeted treatment, identifying high-risk patients as early as possible was also an important factor in reducing mortality [17]. Previous studies compared the differences in various factors between survivors and non-survivors and found that older age, higher sequential organ failure assessment, and D-dimer greater than 1 μg/mL were risk factors for death [11]. More specific indicators meant more operability. In the present study, 26 laboratory markers (Fig. 1) were found to be suitable for identifying high-risk patients at any point during their treatment. The comprehensive usage of these indicators could effectively identify high-risk patients for timely targeted treatments.

Three main limitations should be considered when interpreting these results. First, the results of this study were concluded from 107 patients; thus, indicators with clear values ​​should be adjusted according to their clinical applications, and studies with larger sample sizes are strongly recommended. Second, this was a retrospective study, and the laboratory test results at admission and discharge/death could only be approximated by the first and last tests. Randomized and cohort studies with information of the days from the onset of their symptoms to the first test day are recommended to provide more accurate results. Third, the laboratory findings of COVID-19 infection are approved to be conflicting in different age groups [18], so the results of the present study should be interpreted with caution as the average age of non-survivors significantly differed from the survivors. Despite these limitations, this study presented the temporal changes in systematic laboratory test results of patients with COVID-19, deepening the understanding of this disease and providing actionable laboratory markers to identify high-risk patients.

In summary, we conducted a retrospective study among 82 survivors and 25 non-survivors of adult inpatients with COVID-19 in Wuhan, China, and found that the laboratory test results of 13 and 17 variables were significantly increased and decreased in non-survivors compared with survivors, respectively, both in the first and the last tests. While, the laboratory test results of 18 and 16 variables were significantly increased and decreased in non-survivors compared with survivors, respectively, only in the last tests. The temporal changes of 26 laboratory test variables had obvious differences between these two groups.

## Conclusions

The temporal changes in some laboratory markers of survivors and non-survivors of adult inpatients with COVID-19 were significantly different. A comprehensive usage of these markers might help clinicians identify patients with high risk of COVID-19-associated death or progression from mild to severe disease and provide timely targeted treatment.

## Supplementary Information

**Additional file 1: Table S1.** Selection strategy of laboratory test results with more than five test times that used in the temporal change analysis.

**Additional file 2: Table S2.** The results of laboratory tests of adult inpatients with COVID-19 in Wuhan, China.

**Additional file 3: Fig. S1.** Temporal changes of laboratory makers of adult inpatients with COVID-19 in Wuhan, China. Laboratory test results of each time point is showed in average with 95% confidence intervals. If the lower 95% confidence interval is less than zero, it was replaced with zero. *, ** indicate that the *P* value of variance/welch test in survivors and non-survivors are between 0.001 and 0.05, less than 0.001 in the first test (above the first timescale point), and the last test (above the last timescale point), respectively.

## Data Availability

All the original data are available from the corresponding author as required.
