# Supplementary material for: Temporal changes in laboratory markers of survivors and non-survivors of adult inpatients with COVID-19
Source: BMC Infect Dis. 2020 Dec 11;20:952. doi: 10.1186/s12879-020-05678-0 (PMC7729703; doi:10.1186/s12879-020-05678-0)
Supplement: Supplementary file 1 — Additional file 1: Table S1. Selection strategy of laboratory test results with more than five test times that used in the temporal change analysis. [file 12879_2020_5678_MOESM1_ESM.docx]

Table S1. Selection strategy of laboratory test results with more than five test times that used in the temporal change analysis.

| Total times of laboratory test | First timescale point | Second timescale point | Third timescale point | Fourth time scale point | Fifth timescale point |
| --- | --- | --- | --- | --- | --- |
| 5 | 1 | 2 | 3 | 4 | 5 |
| 6 | 1 | 2 | 3 | 5 | 6 |
| 7 | 1 | 2 | 4 | 5 | 7 |
| 8 | 1 | 2 | 4 | 6 | 8 |
| 9 | 1 | 2 | 5 | 7 | 9 |
| 10 | 1 | 3 | 5 | 8 | 10 |
| 11 | 1 | 3 | 6 | 8 | 11 |
| 12 | 1 | 3 | 6 | 9 | 12 |
| 13 | 1 | 3 | 7 | 10 | 13 |
| 14 | 1 | 4 | 7 | 11 | 14 |
| 15 | 1 | 4 | 8 | 11 | 15 |
| 16 | 1 | 4 | 8 | 12 | 16 |
| 17 | 1 | 4 | 9 | 13 | 17 |
| 18 | 1 | 5 | 9 | 14 | 18 |
| 19 | 1 | 5 | 10 | 14 | 19 |
| 20 | 1 | 5 | 10 | 15 | 20 |
| 21 | 1 | 5 | 11 | 16 | 21 |
| 22 | 1 | 6 | 11 | 17 | 22 |
| 23 | 1 | 6 | 12 | 17 | 23 |
| 24 | 1 | 6 | 12 | 18 | 24 |
| 25 | 1 | 6 | 13 | 19 | 25 |
| 26 | 1 | 7 | 13 | 20 | 26 |
| 27 | 1 | 7 | 14 | 20 | 27 |
