# Supplementary material for: Temporal changes in laboratory markers of survivors and non-survivors of adult inpatients with COVID-19
Source: BMC Infect Dis. 2020 Dec 11;20:952. doi: 10.1186/s12879-020-05678-0 (PMC7729703; doi:10.1186/s12879-020-05678-0)
Supplement: Supplementary file 2 — Additional file 2: Table S2. The results of laboratory tests of adult inpatients with COVID-19 in Wuhan, China. [file 12879_2020_5678_MOESM2_ESM.docx]

Table S2. The results of laboratory tests of adult inpatients with COVID-19 in Wuhan, China

| Laboratory variable | Survivors | | | | Non-survivors | | | |
| --- | --- | --- | --- | --- | --- | --- | --- | --- |
|  | N | Average | Lower 95% CI | Upper 95% CI | N | Average | Lower 95% CI | Upper 95% CI |
| Lymphocyte count | 74 | 1.04 | 0.92 | 1.16 | 24 | 0.62 | 0.52 | 0.72 |
| Lymphocyte percentage | 74 | 20.82 | 17.77 | 23.88 | 24 | 6.70 | 5.36 | 8.03 |
| Albumin | 70 | 36.60 | 35.14 | 38.07 | 21 | 30.27 | 28.45 | 32.08 |
| Neutrophil count | 74 | 4.74 | 3.96 | 5.51 | 24 | 9.77 | 7.53 | 12.00 |
| Neutrophil percentage | 74 | 71.39 | 67.95 | 74.83 | 24 | 88.14 | 85.75 | 90.53 |
| Platelet distribution | 74 | 16.18 | 16.09 | 16.28 | 24 | 16.63 | 16.42 | 16.83 |
| Lactate dehydrogenase | 68 | 276.57 | 238.58 | 314.55 | 20 | 465.15 | 388.57 | 541.73 |

95% CI = 95% confidence interval.
