# Supplementary figures and images for: Temporal changes in laboratory markers of survivors and non-survivors of adult inpatients with COVID-19
Source: BMC Infect Dis. 2020 Dec 11;20:952. doi: 10.1186/s12879-020-05678-0 (PMC7729703; doi:10.1186/s12879-020-05678-0)

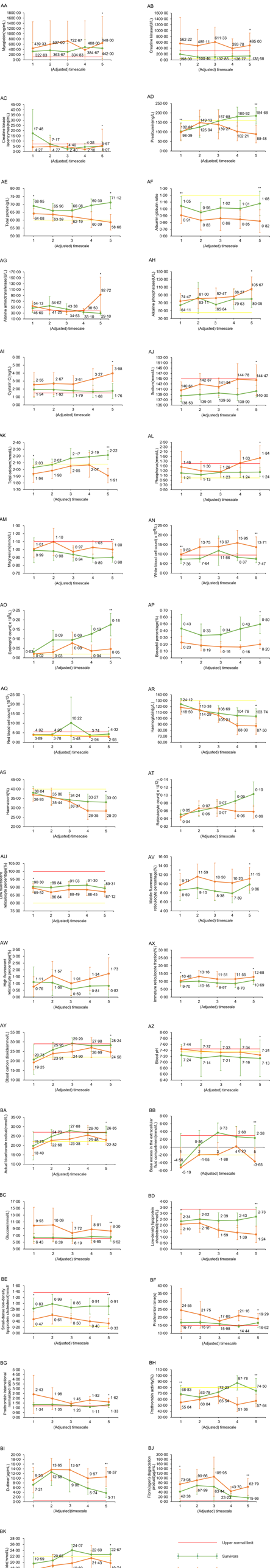

Supplement: Supplementary file 3 — Additional file 3: Fig. S1. Temporal changes of laboratory makers of adult inpatients with COVID-19 in Wuhan, China. Laboratory test results of each time point is showed in average with 95% confidence intervals. If the lower 95% confidence interval is less than zero, it was replaced with zero. *, ** indicate that the P value of variance/welch test in survivors and non-survivors are between 0.001 and 0.05, less than 0.001 in the first test (above the first timescale point), and the last test (above the last timescale point), respectively. [file 12879_2020_5678_MOESM3_ESM.pdf]
